# Neuroinflammation underlies the development of social stress induced cognitive deficit in male sickle cell mice

**DOI:** 10.3389/ebm.2024.10361

**Published:** 2024-11-19

**Authors:** S’Dravious A. DeVeaux, Sofiya Vyshnya, Katherine Propsom, Oluwabukola T. Gbotosho, Asem S. Singh, Robert Z. Horning, Mihika Sharma, Anil G. Jegga, Liang Niu, Edward A. Botchwey, Hyacinth I. Hyacinth

**Affiliations:** ^1^ The Wallace H. Coulter Department of Biomedical Engineering, Georgia Tech and Emory, Atlanta, GA, United States; ^2^ Petit Institute of Bioengineering and Biosciences, Georgia Institute of Technology, Atlanta, GA, United States; ^3^ Department of Neurology and Rehabilitation Medicine, University of Cincinnati College of Medicine, Cincinnati, OH, United States; ^4^ Division of Biomedical Informatics, Department of Pediatrics, Cincinnati Children’s Hospital Medical Center, University of Cincinnati College of Medicine Cincinnati, Cincinnati, OH, United States; ^5^ Department of Environmental and Public Health Sciences, University of Cincinnati College of Medicine, Cincinnati, OH, United States

**Keywords:** sickle cell disease, neuroinflammation, cognitive function, social stress, minocycline

## Abstract

Cognitive deficit is a debilitating complication of sickle cell disease (SCD), with a multifactorial etiopathogenesis. Here we show that neuroinflammation and dysregulation in lipidomics and transcriptomics profiles are major underlying mechanisms of social stress-induced cognitive deficit in SCD. Male Townes sickle cell (SS) mice and controls (AA) were exposed to social stress using the repeat social defeat (RSD) paradigm concurrently with or without treatment with minocycline. Mice were tested for cognitive deficit using novel object recognition and fear conditioning tests. SS mice exposed to RSD without treatment had worse performance on cognitive tests compared to SS mice exposed to RSD with treatment or to AA controls, irrespective of their RSD or treatment disposition. Additionally, compared to SS mice exposed to RSD with treatment, SS mice exposed to RSD without treatment had significantly more cellular evidence of neuroinflammation coupled with a significant shift in the differentiation of neural progenitor cells towards astrogliogenesis. Additionally, brain tissue from SS mice exposed to RSD was significantly enriched for genes associated with blood-brain barrier dysfunction, neuron excitotoxicity, inflammation, and significant dysregulation in sphingolipids important to neuronal cell processes. We demonstrate in this study that social stress induces cognitive deficit in SS mice, concurrently with neuroinflammation and lipid dysregulation.

## Impact statement

We show for the first time that neuroinflammation along with changes in the brain lipidome and transcriptome, are underlying biological mechanisms contributing to the development and potentially progression of cognitive impairment in SCD mice. These findings also provide for the first time, a potential mechanistic basis for an earlier reported observation of a higher likelihood of having lower intelligence quotient scores among children with sickle cell disease exposed to social stress in the form of low parental socioeconomic status.

## Introduction

Sickle cell disease (SCD) is a common inherited blood disorder that affects approximately 100,000 Americans and millions more worldwide [1, 2]. SCD is caused by a point mutation in the gene for the β-globin subunit of hemoglobin. This mutation causes the hemoglobin to polymerize in conditions of low oxygen tension, causing the red blood cells (RBCs) to assume a sickle morphology [2, 3]. Sickle RBCs are more fragile and prone to hemolysis, leading to anemia; the resulting free heme also initiates and propagates an inflammatory cascade that leads to vaso-occlusion [2, 3], and end organ damage [4].

The cerebrovascular effects of SCD include silent cerebral infarctions (SCIs) found in ∼39% of children by 18 years of age and >50% of adults by 30 years of age, stroke, cerebral macro- and microvascular abnormalities [5]. Strokes and SCIs have been linked to cognitive impairment in SCD. However, recent studies have found cognitive dysfunction in children [6–8] and adults [9, 10] even in the absence of MRI-detectable cerebral injury. Children with SCD typically have lower full-scale IQ scores, poorer academic achievement, and impaired processing speed [6, 7]. Similarly, adults with SCD exhibit impairments in processing speed, working memory, global cognitive function, and executive function.

The mechanism underlying cognitive impairment in SCD is not well understood, and one possibility is that individuals with SCD are hypersensitive to social stressors (to which individuals with SCD are exposed), which interact with biological factors leading to the development of cognitive deficit. Individuals with SCD often belong to lower socioeconomic classes with associated lower family educational attainment and income. The impact of social stress on cognitive function in SCD was recently demonstrated by several studies [5, 11–13]. In a study by King *et al.*, they reported that social stressors in the form of lower parental education levels and lower family income – had a similar albeit slightly more severe impact on cognitive function compared to biological factors – such as the presence of SCI, anemia, and age [14, 15]. Studies in the general human population and in non-sickle cell mouse models have shown a link between social stress and neuroinflammation. The functional effects of neuroinflammation on the brain include the development of cognitive impairment as well as neuropsychological abnormalities, such as anxiety and depression [16]. As well as learning and memory impairments [17–19]. Hence, neuroinflammation may be a possible mechanism for stress-induced cognitive abnormalities in SCD.

Neuroinflammation is also mediated by multiple factors, including sphingolipids and genetics. Sphingolipids are a class of bioactive lipids that participate in cell signaling. In the brain, sphingolipids modulate cytokine release and astroglia activation [20]. Studies have shown that imbalances in the sphingolipid metabolism and distribution of lipids in the brain are associated with impaired memory and learning in both humans and animal models [21–26]. Furthermore, enzymes in the sphingolipid pathway – such as sphingosine kinases, sphingosine-1-phosphate lyase, and sphingomyelinases – are involved in synaptic communication, learning, and memory, as well as in the regulation of other enzymes (e.g., COX2) that synthesize both pro-inflammatory and anti-inflammatory lipid mediators. Changes in the enzymatic activity have been implicated in the development of neuroinflammation and neurodegenerative diseases, such as Alzheimer’s and amyotrophic lateral sclerosis [27, 28].

We have previously shown that aging and neuroinflammation contribute to cognitive impairment in the SCD mouse model [29]. In the present study, we demonstrate the role of sociological stress in cognitive and neurobehavioral deficits in SCD and show that neuroinflammation is a likely underlying mechanism. We used the repeat social defeat (RSD) paradigm as a social stress model, as previously described [17]. Our overall hypothesis is that *stress-related cognitive impairment in sickle cell disease is mediated by neuroinflammation and inimical changes in the brain lipidomic and transcriptomic profiles compared to controls.* Furthermore, treatment with minocycline, an “anti-neuroinflammatory” drug, during RSD exposure in mice will reduce neuroinflammation and improve cognitive and behavioral function.

## Materials and Methods

### Animals

We used humanized Townes sickle cell mouse model (SS or HbSS) and humanized control mice (AA or HbAA) [30]. Mice were provided food and water *ad libitum*, housed in a 12-h light and dark cycle, and their health statuses were monitored closely throughout the study. All experiments were approved by the Institutional Animal Use Committee at Emory University.

### Study design and overall methods

This study aims to examine the mechanism underlying the development of cognitive deficit in SCD with exposure to social stress, by using the repeat social defeat (RSD) paradigm in male SS mice. RSD was carried out by introducing a male intruder mouse (an aggressor) into an established cage containing three 6-month-old male SS mice (N = 10) or AA mice (N = 10) every day for 2 hours (5–7 p.m.) for six consecutive days. Age- and sex-matched control [SS (N = 10) and AA (N = 10) mice] cages were set up but without aggressor mice. On the seventh day, mice were tested for cognitive/behavioral deficit using novel object recognition (NOR) and fear conditioning (FC) test paradigms. Except for the aggressors, all mice used were Townes humanized SS and AA mice.

To test the hypothesis that neuroinflammation is an underlying mechanism, a second cohort of SS (N = 35) and AA (N = 32) mice were randomly assigned to receive oral (administered in drinking water) minocycline treatment (90 mg/kg) or placebo (plain drinking water). We assigned 2 mice to the minocycline plus stress arm (due to the potential for injury and mortality from the RSD paradigm) for every 1 mouse assigned to the other arms, from each genotype group. Mice within each treatment arm were randomly assigned to RSD exposure or no RSD exposure. Minocycline treatment was started 1 day prior to the day of commencing RSD and co-terminated on the same day as the final RSD session. The minocycline dose was kept constant by adjusting the amount administered daily, using the water/drug consumption from the previous day. Cognitive/behavioral testing was performed as before, starting the next day after day 6 of RSD and day 7 of treatment.

In both experiments, the mice were randomized to histological analysis or molecular (bulk RNA sequencing and lipidomics) studies and sacrificed 1-2 days after the completion of behavioral testing. Their brains were extracted for the assigned analysis. Cellular evidence of neuroinflammation in the hippocampus/dentate gyrus was determined using immunohistochemistry to quantify peripheral immune cell infiltrates:CD45^+^ (bone marrow-derived microglia), CD3^+^ (T-cell density), B220^+^ (B-cell density), and Iba1^+^ (activated microglia).

A more detailed description of the study methods is in the methods section of the Online Supplementary Material. Data reporting is in accordance with the ARRIVE guidelines.

## Results

All studies were conducted using male mice. Experimental groups in this study are defined as follows: humanized control mice (AA) and Townes sickle (SS) mice, and also denote animals not exposed to stress or treated with minocycline; AA + RSD and SS + RSD denote mice exposed to stress (RSD); AA + minocycline and SS + minocycline denote mice not exposed to RSD but treated with minocycline; AA + RSD + minocycline and SS + RSD + minocycline denote mice treated with minocycline 1 day prior to and during exposure to stress.

**FIGURE 1:**
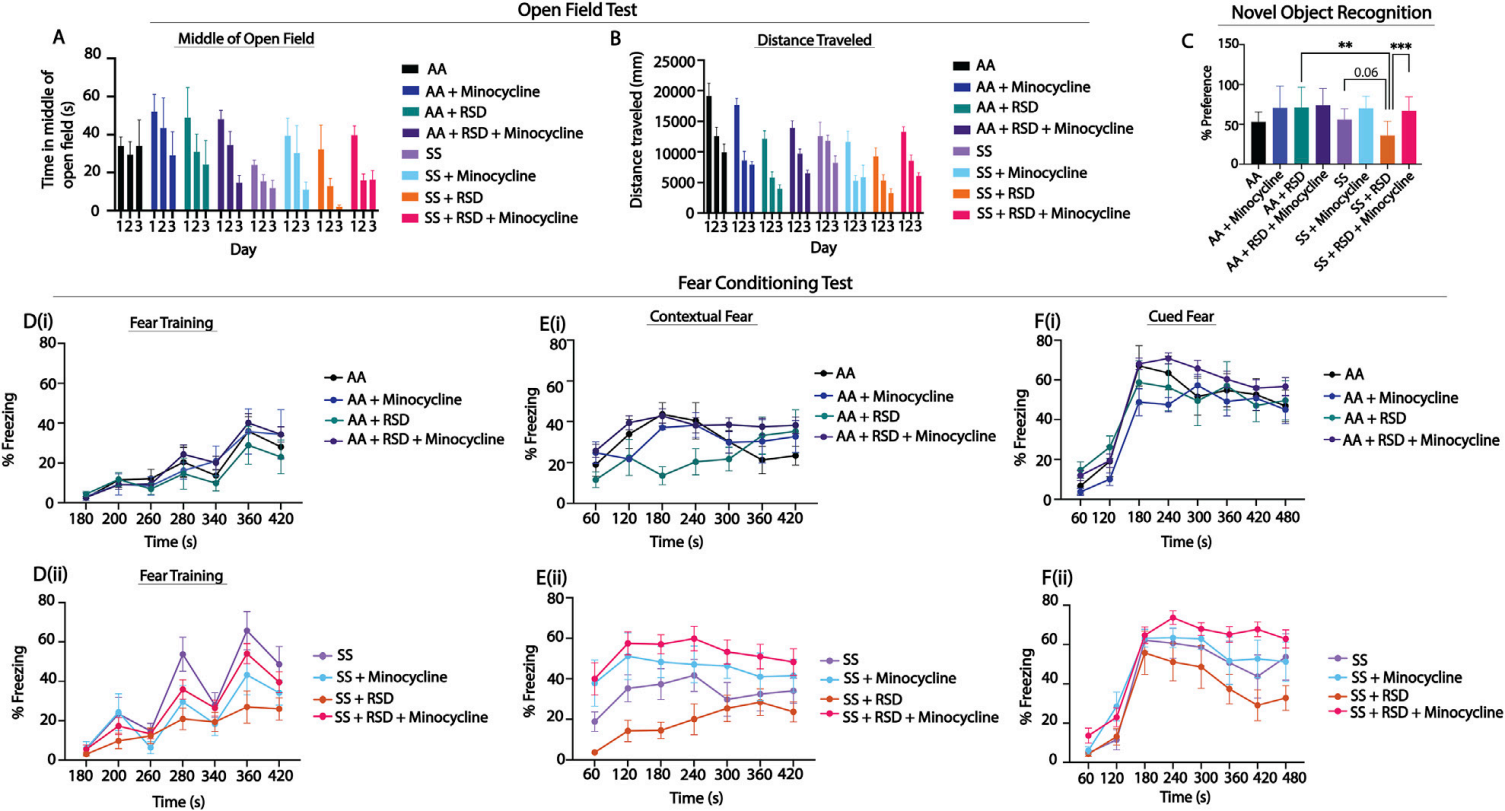
Sickle mice display significant cognitive and neurobehavioral deficits under stress compared to control mice. **(A, B)** illustrate the results for the open field test, depicting distance traveled through the open field arena and time spent in the middle of the arena. Statistical comparisons were conducted using a two-way ANOVA (using a mixed effect model) with Tukey’s multiple comparisons test. **(C)** depicts preference for the novel object in the NOR test; analysis was with a one-way ANOVA with multiple comparisons (using Fisher’s LSD) conducted to compare object preference between groups. **(D, F)** illustrate results for the fear conditioning studies (a measure of associative memory). **(Di,ii)** represents the training phase, where mice acquired a fear response to an 85 dB tone that was paired with a shock through classical conditioning. **(Ei,ii)** and **(Fi,ii)** test the strength of the animals’ conditioned fear response by observing freezing behavior (indicative of fear) after being placed in the same environment where the shock had been administered during the learning phase or after hearing the 85 db tone that was associated with the shock, respectively. Freezing behavior was compared between groups using a two-way ANOVA (using a mixed effect model) with Holm-Sidak’s adjustment for multiple comparisons for the contextual and cued fear assessments. All groups (AA and SS) were analyzed together. The figures **(E, F)** were split based on genotype to enhance the clarity of the presentation of the result. AA mice (n = 7–9), AA + RSD mice (n = 6–9), AA + minocylcine mice (n = 6), AA + RSD + minocycline (n = 13–21), SS mice (n = 6–7), SS + RSD mice (n = 6–10), SS + minocycline mice (n = 7), SS + RSD + minocycline mice (n = 15–22). *p < 0.05, **p < 0.01, ***p < 0.001. Data are presented as *mean ± SEM*.

Figure 1 shows comparison of the groups on measures of anxiety (open-field test) and cognitive function (percent preference or freezing). In Figures 1A, B, overall, we see that SS mice that were not exposed to RSD or drug treatment showed more evidence of anxiety compared to AA mice that were not exposed to RSD, indicated by the shorter distance traveled (Figure 1A, day 1: 19,116 mm in AA vs. 12,593 mm in SS, n.s.) and relatively shorter time spent in the middle of the open field (Figure 1B, day 1: 33.94 s in AA vs. 24.06 s in SS, n.s.). Furthermore, SS mice exposed to RSD showed more evidence of anxiety compared to AA mice exposed to RSD (distance traveled: day 1: 12,593 mm in SS vs. 9,274 cm in SS + RSD; time in the middle of the open field: day 1: 24.1 s in SS vs. 32.3 s in SS + RSD) or to SS or AA mice. Treatment with minocycline abrogated the development of anxiety in SS mice exposed to RSD (distance traveled: day 1: 13,276 mm in SS + RSD + Minocycline vs. 9,274 mm in SS + RSD; time in open field: day 1: 39.6 s in SS + RSD + Minocycline vs. 32.28 s in SS + RSD: Figures 1A, B).

Furthermore, evaluation of hippocampus-dependent non-associative as well as associative learning and memory, was carried out using NOR and fear conditioning, respectively. In the NOR test, (Figure 1C), SS and AA mice had similar percent preference (56.1 ± 14% vs. 53.1 ± 13%), indicating comparable non-associative memory. However, SS + RSD mice showed some evidence of cognitive impairment as demonstrated by lower percent preference (36.1 ± 18% SS + RSD vs. 56.1 ± 14% SS, *p* = 0.06) compared to SS mice, indicating impaired non-associative memory function. Additionally, we also noted that SS + RSD + minocycline mice had significantly higher percent preference (67.1 ± 18% vs. 36.1 ± 18%, *p* = 0.0007) compared to SS + RSD mice, suggesting that minocycline treatment led to a sparing of non-associative memory in the treated mice despite exposure to RSD. On the other hand, in the AA group, neither stress nor minocycline treatment were associated with significant changes in cognitive function.

Likewise, the fear conditioning tests (Figures 1D–F) showed that overall, sickle and non-sickle mice, irrespective of treatment or exposure disposition, trained similarly during the acquisition phase (Figure 1D). As shown in Figure 1E, RSD exposure resulted in significant impairment in contextual (associative) fear memory (evidenced by significantly lower percent freezing) in SS + RSD mice, compared to SS mice. As in Figures 1C, E shows that SS + RSD + minocycline mice had significantly better contextual fear memory, compared to SS + RSD mice, p = 0.025 to p < 0.0001 across all time points except at 240 s. The abnormal contextual fear memory indicates possible molecular disturbance and/or “overt or covert” lesion of the amygdala resulting from exposure to RSD and its abrogation by minocycline treatment.

Similarly, on cued fear testing (Figure 1F), there was no significant difference in response from the unperturbed AA or SS mice. However, SS + RSD mice showed significant impairment in hippocampus-mediated cued (associative) fear memory compared to SS + RSD + minocycline mice (p = 0.016 to <0.0001) across different time points. In contrast, neither RSD nor minocycline had significant effects on cognitive function among the AA genotypes.

**FIGURE 2:**
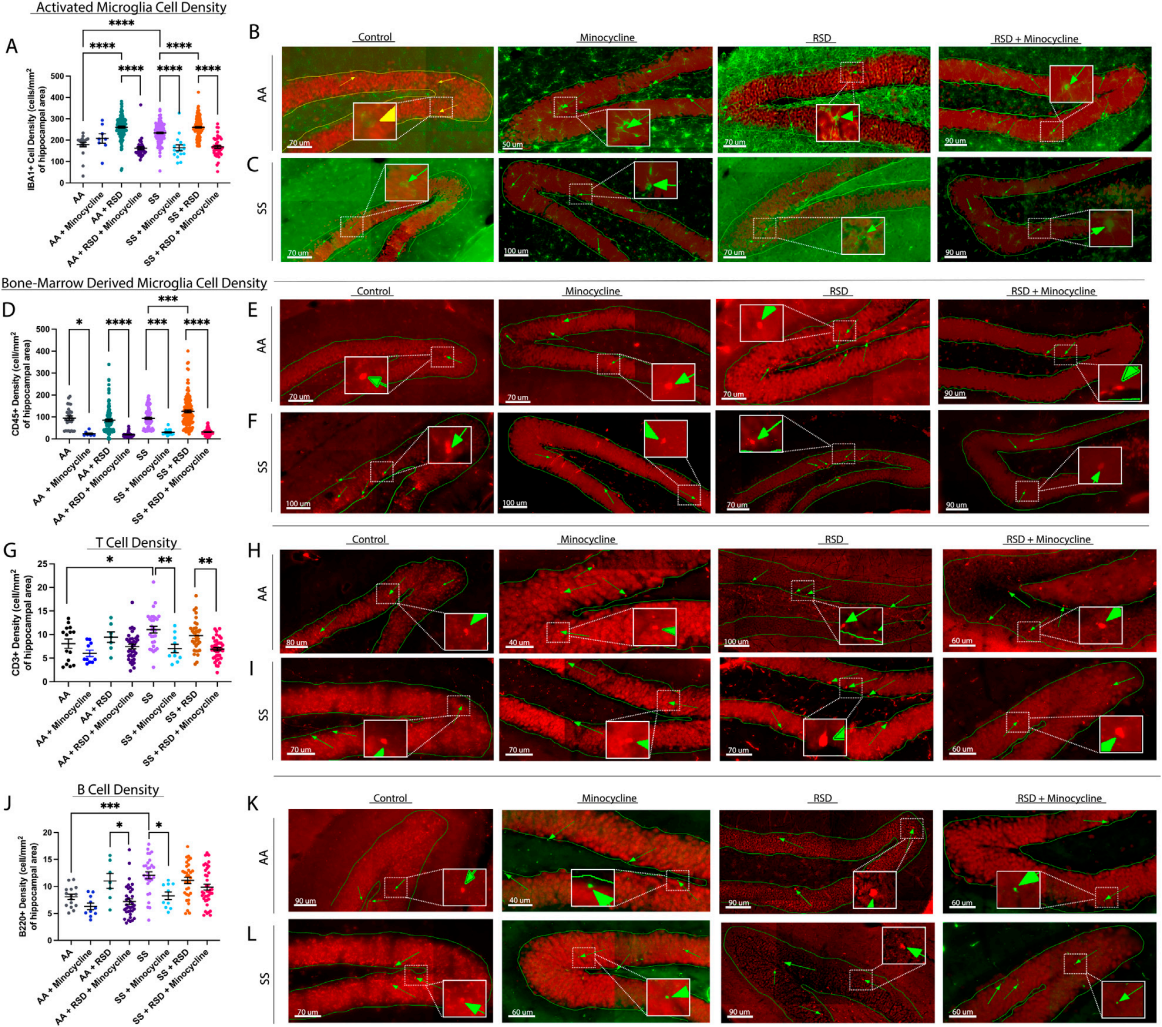
Sickle cell mice exposed to RSD have a higher density of activated microglia and B and T cell infiltrates in the hippocampus while minocycline reduces the density of immune cell infiltrates. **(A)** IBA-1+ activated microglia cell density. **(B)** Immunohistochemistry images showing IBA-1+ activated microglia in AA control mice (n = 10 sections), AA mice treated with minocycline (n = 8 sections), AA mice exposed to RSD (n = 27 sections), and AA mice exposed to RSD and treated with minocycline (n = 40 sections). **(C)** Immunohistochemistry images showing IBA-1+ activated microglia in SS control mice (n = 22 sections), SS mice treated with minocycline (n = 18 sections), SS mice exposed to RSD (n = 41 sections), and SS mice exposed to RSD and treated with minocycline (n = 49 sections). **(D)** CD45^+^ “bone-marrow-derived” microglia cell density. **(E)** Immunohistochemistry images showing CD45^+^ bone-marrow microglia in AA control mice (n = 10 sections), AA mice treated with minocycline (n = 8 sections), AA mice exposed to RSD (n = 27 sections), and AA mice exposed to RSD and treated with minocycline (n = 40 sections). **(F)** Immunohistochemistry images showing CD45^+^ bone-marrow microglia in SS control mice (n = 22 sections), SS mice treated with minocycline (n = 18 sections), SS mice exposed to RSD (n = 41 sections), and SS mice exposed to RSD and treated with minocycline (n = 49 sections). **(G)** B220 + B cell density. **(H)** Immunohistochemistry images showing B220 + B cells in AA control mice (n = 16 sections), AA mice treated with minocycline (n = 10 sections), AA mice exposed to RSD (n = 8 sections), and AA mice exposed to RSD and treated with minocycline (n = 36 sections). **(I)** Immunohistochemistry showing B220+ B cells in SS control mice (n = 32 sections), SS mice treated with minocycline (n = 10 sections), SS mice exposed to RSD (n = 32 sections), and SS mice exposed to RSD and treated with minocycline (n = 40 sections). **(J)** CD3^+^ T cell density. **(K)** Immunohistochemistry images showing CD3^+^ T cells in AA control mice (n = 16 sections), AA mice treated with minocycline (n = 10 sections), AA mice exposed to RSD (n = 8 sections), and AA mice exposed to RSD and treated with minocycline (n = 36 sections). **(L)** Immunohistochemistry images showing CD3^+^ T cells in SS control mice (n = 32 sections), SS mice treated with minocycline (n = 10 sections), SS mice exposed to RSD (n = 32 sections), and SS mice exposed to RSD and treated with minocycline (n = 40 sections). Cell density was compared between groups with a one-way ANOVA and Fisher’s LSD multiple comparisons test. *p < 0.05, **p < 0.01, ***p < 0.001. Data are presented as mean ± SEM. The “n” in brackets after each group represents the total number of hippocampal brain tissue sections evaluated.

Next, we evaluated the density of peripheral immune cell infiltrates (known from here on as CD45^+^ “bone-marrow derived” microglia [BMDM]), Iba1^+^ activated microglia (activation state determined based on morphological features), CD3^+^ T cells, and B220^+^ B cells in the hippocampus/dentate gyrus (DG). We focused on the hippocampus/DG because of its critical role in cognitive function as well as adult neurogenesis [31]. In Figure 2A, we show that overall, SS mice had a higher density of activated microglia (233.6 ± 44.6 cells/mm^2^ vs. 179.3 ± 47.0 cells/mm^2^, *p* ≤ 0.0001) compared to AA mice. Furthermore, SS + minocycline mice had a significantly lower density of activated microglia (164.4 ± 56.6 cells/mm^2^ vs. 233.6 ± 44.6 cells/mm^2^, *p* ≤ 0.0001) compared to SS mice. Similarly, SS + RSD mice had a significantly higher density of activated microglia (260.2 ± 44.3 cells/mm^2^ vs. 233 ± 44.6 cells/mm^2^, *p* ≤ 0.0001) compared to SS mice. Finally, we observed that SS + RSD + minocycline mice showed a 35% decrease (p ≤ 0.0001) in activated microglia density compared to SS + RSD mice. As shown in Figure 2A, the results for the comparison within the AA groups were similar to those described for the SS mice. Figures 2B, C are representative images. Additionally, except for the control (non-perturbed group), there were no significant differences between the AA and SS mice based on RSD exposure or minocycline treatment.

Results of the examination of the contribution of peripheral immune cell (CD45^+^) infiltrate to the observed cognitive deficit and impact of minocycline treatment are shown in Figure 2D. There was no significant difference between SS and AA mice with respect to the density of CD45^+^ BMDM cells in the hippocampus/DG. However, both AA + RSD and SS + RSD mice had a significantly higher density of CD45^+^ BMDM compared to their non-perturbed controls, with SS + RSD mice having about 1.5-fold (p < 0.0001) more CD45^+^ BMDM than AA + RSD mice. Additionally, we found a 75% (p = 0.013) and 77% (p < 0.0001) decrease in CD45^+^ BMDM density in AA mice and AA + RSD treated with minocycline, respectively, compared to their non-treated controls. Similarly, stress exposure significantly increased the density of CD45^+^ BMDM in SS mice (from 93.7 ± 45.8 cells/mm^2^ to 125.4 ± 70.3 cells/mm^2^, p = 0.0004), while minocycline treatment reduced the density in unperturbed SS mice by 3.1-fold (p = 0.0002) and in SS + RSD mice by 3.9-fold (p < 0.0001). Figures 2E, F are representative images. Furthermore, we quantified the density of B cells (B220^+^) and T cells (CD3^+^) as shown in Figures G. Notably, AA + RSD mice had a significantly higher density of B cells (11.0 ± 3.7 cells/mm^2^ vs. 7.2 ± 3.0 cells/mm^2^, p = 0.037) compared to AA + RSD + minocycline mice. There was also a slight decrease in B cell density in AA + minocycline mice compared to AA mice, though not significant. This suggests that minocycline may be suppressing B-cell-mediated neuroinflammation by limiting peripheral immune cell infiltration into the brain. Furthermore, when compared to AA mice, SS mice had a significantly higher density of B cells (12.1 ± 3.4 cells/mm^2^ vs. 8.1 ± 1.9 cells/mm^2^, p = 0.0009), and when SS mice were treated with minocycline, the density of B cells decreased by 31% (p = 0.012). Figures 2H, I are representative images. Surprisingly, exposure of SS mice to RSD with or without minocycline treatment did not result in a significant change in B cell density, contrary to our observation in AA mice. This result suggests that B cell infiltration might play a smaller role in RSD-induced neuroinflammation as an underlying mechanism for the development of cognitive deficits in SCD.

Further analysis, as shown in Figure 2J, indicates that SS mice had significantly higher T cell density (11.1 ± 3.8 cells/mm2 vs. 8.1 ± 3.8 cells/mm2, p = 0.032) compared to AA mice. Minocycline treatment decreased T cell density in AA mice by 26% (p = 0.032), while T cell density in AA + RSD and AA + RSD + minocycline mice was similar. Among sickle cell groups, minocycline treatment resulted in a 1.6-fold (p = 0.005) reduction in T cell density in unperturbed SS mice and about a 30% reduction in SS + RSD mice compared to their untreated controls. Figures 2K, L are representative images of the plot. However, there was no significant difference in T cell density between SS mice and SS + RSD mice. This indicates a possible but slightly lesser role for T cells in cognitive impairment in SCD in the setting of exposure to social stress.

**FIGURE 3:**
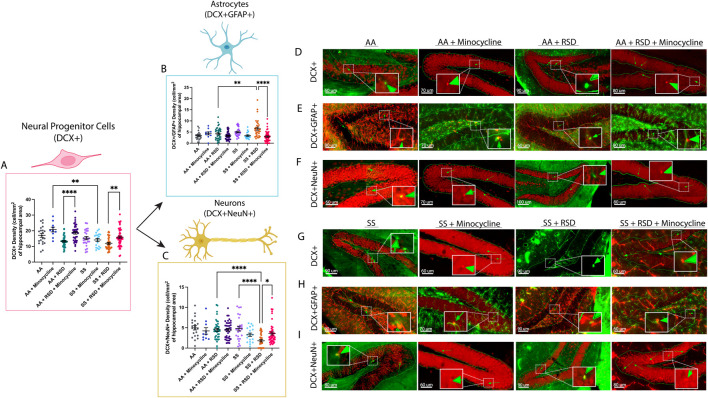
Sickle cell mice exposed to RSD have a higher density of DCX^+^GFAP^+^ astrocytes while having decreased densities of DCX^+^ neural progenitor cells and DCX^+^NeuN^+^ neurons in the hippocampus. **(A)** DCX^+^ neural progenitor cell density. **(B)** DCX^+^GFAP^+^ astrocyte cell density. **(C)** DCX^+^NeuN^+^ neuron cell density **(D)** Immunohistochemistry images showing DCX^+^ neural progenitor cells in AA control mice (n = 21 sections), AA mice treated with minocycline (n = 10 sections), AA mice exposed to RSD (n = 40 sections), and AA mice exposed to RSD and treated with minocycline (n = 41 sections). **(E)** Immunohistochemistry images showing DCX^+^GFAP^+^ astrocytes in AA control mice (n = 21 sections), AA mice treated with minocycline (n = 10 sections), AA mice exposed to RSD (n = 40 sections), and AA mice exposed to RSD and treated with minocycline (n = 41 sections). **(F)** Immunohistochemistry images showing DCX^+^NeuN^+^ neurons cells in AA control mice (n = 21 sections), AA mice treated with minocycline (n = 10 sections), AA mice exposed to RSD (n = 40 sections), and AA mice exposed to RSD and treated with minocycline (n = 41 sections). **(G)** Immunohistochemistry images showing DCX^+^ neural progenitor cells in SS control mice (n = 23 sections), SS mice treated with minocycline (n = 20 sections), SS mice exposed to RSD (n = 40 sections), and SS mice exposed to RSD and treated with minocycline (n = 49 sections). **(H)** Immunohistochemistry images showing DCX^+^GFAP^+^ astrocytes in SS control mice (n = 23 sections), SS mice treated with minocycline (n = 20 sections), SS mice exposed to RSD (n = 40 sections), and SS mice exposed to RSD and treated with minocycline (n = 49 sections). **(I)** Immunohistochemistry images showing DCX^+^NeuN^+^ neurons cells in SS control mice (n = 23 sections), SS mice treated with minocycline (n = 20 sections), SS mice exposed to RSD (n = 40 sections), and SS mice exposed to RSD and treated with minocycline (n = 49 sections). Statistical comparisons were performed with a one-way ANOVA with Fisher’s LSD multiple comparisons test. *p < 0.05, **p < 0.01, ****p < 0.0001. Data are presented as mean ± SEM. ****p < 0.0001. The “n” in brackets after each group represents the total number of hippocampal brain tissue sections evaluated.

Given the reported role of neurogenesis in social stress-induced cognitive deficits [18, 32] due to neuroinflammation, we quantified and compared the densities of neural progenitor cells (NPCs; DCX^+^), adult-born neurons (DCX^+^NeuN^+^), and “newly formed” astrocytes (DCX^+^GFAP^+^) in the dentate gyrus and reported our findings in Figure 3. As shown in Figure 3A, we observed a higher density of NPCs in AA + minocycline mice (20.8 ± 4.8 cells/mm^2^ vs. 17.1 ± 5.7 cells/mm^2^) compared to AA mice, while the NPC density was lower among AA + RSD mice (13.3 ± 3.6 cells/mm^2^, p = 0.06) compared to AA mice. Additionally, AA + RSD mice had significantly lower NPC density compared to AA + RSD + minocycline mice (18.7 ± 5.3 cells/mm^2^, p < 0.0001). We also observed that SS mice had slightly lower NPC density compared to AA mice and that minocycline treatment significantly reduced NPC density in SS mice compared to treated AA mice (p = 0.006). Furthermore, as seen in the AA groups, SS + RSD mice had a significantly lower NPC density (11.9 ± 3.9 cells/mm^2^ vs.15.8 ± 5.6 cells/mm^2^, *p* = 0.005) compared to SS + RSD + minocycline mice. Figures 3D–F are representative images. We also noted that SS + RSD + minocycline mice have essentially the same NPC density as SS mice, indicating that minocycline might be limiting the gliogenic shift that seems to result from exposure to RSD (see Figure 3B; Supplementary Figure S1), leading to the development of cognitive deficit.

Furthermore, as shown in Figure 3B, SS mice had more newly generated astrocytes (5.0 ± 1.9 cells/mm^2^ vs. 3.3 ± 1.9 cells/mm^2^) than AA mice, and exposure to RSD increased the density of new astrocytes in both AA and SS mice, though not significantly when compared to their respective unperturbed controls. However, there was a 35% increase (p = 0.0011) in the density of new astrocytes in SS + RSD mice compared to AA + RSD. Interestingly, minocycline treatment reduced the density of new astrocytes in SS mice and AA + RSD mice, with the most significant decrease of 55% observed in the treated SS + RSD mice. Taken together, this indicates that the exposure to RSD alone might be shifting the differentiation of NPCs towards astrocytes. And that treatment with minocycline reduces that shift as seen in the SS + RSD + minocycline and AA + RSD + minocycline mice when compared to their untreated but stressed counterparts.

Results of the quantification of the density of adult-born neurons (DCX^+^NeuN^+^) are shown in Figure 3C; Supplementary Figure S1. Overall, we show that AA mice, irrespective of treatment or RSD status, had similar adult-born neuron densities. Among the SS mice, SS + RSD mice had significantly lower adult-born neuron density compared with SS mice (1.8 ± 1.7 cells/mm^2^ vs. 4.8 ± 2.9 cells/mm^2^, p < 0.0001). In contrast to similarly stressed AA mice, SS mice also displayed a more pronounced effect of RSD exposure on adult-born neuron density, with a 2.5-fold (p < 0.0001) decrease. However, treatment of SS + RSD mice with minocycline resulted in a 50% (p = 0.0031) increase in adult-born neurons. Supplementary Figure S1 provides the percentage distribution of these cells (DCX^+^GFAP^+^ and DCX^+^NeuN^+^) as a percentage of the total NPCs (DCX^+^) counted.

**FIGURE 4:**
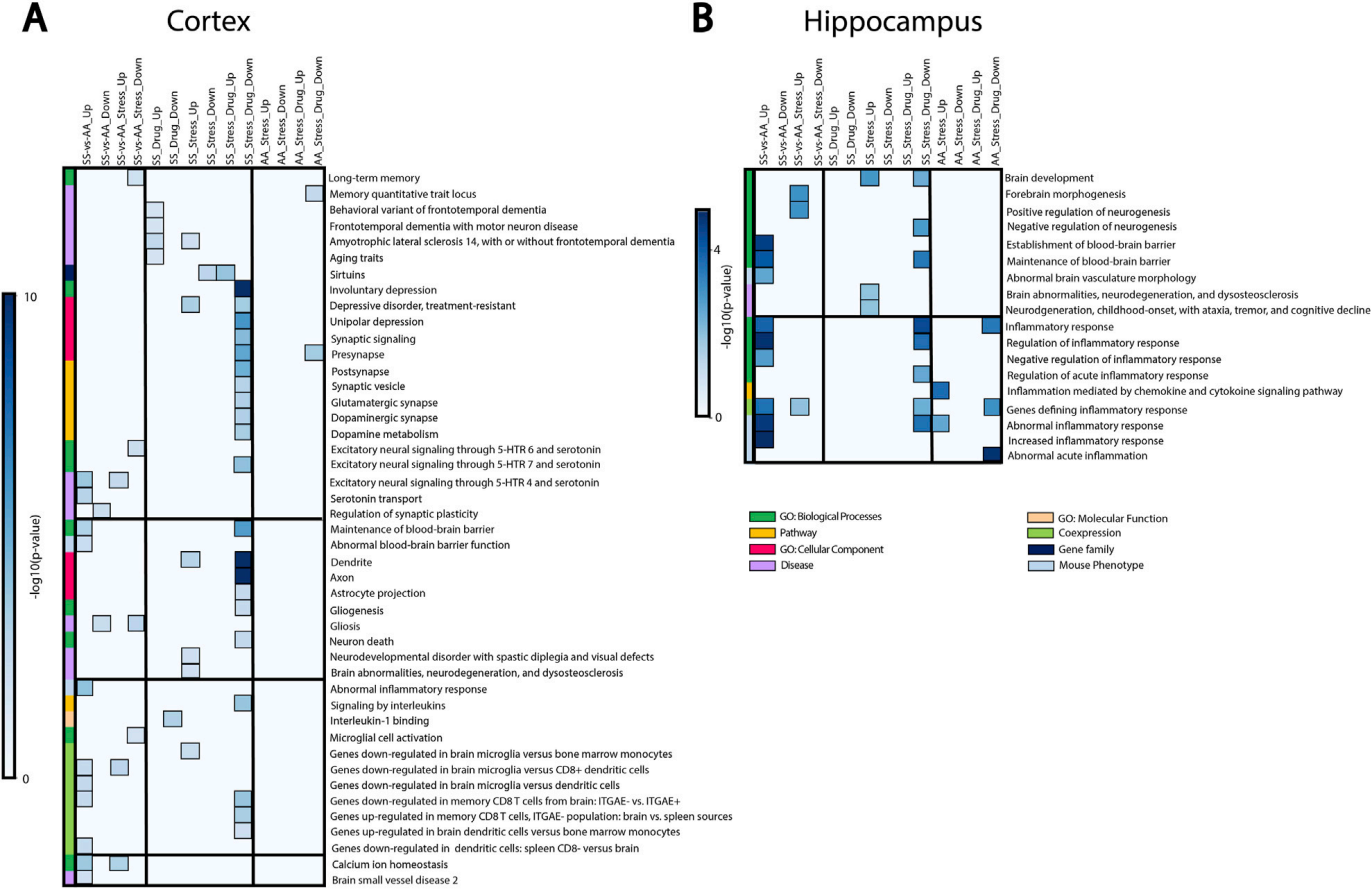
Gene set enrichment analysis showing how RSD and minocycline affect pathways, biological processes, and diseases related to cognitive function, brain development, and inflammation. SS-vs-AA_UP: genes enriched in control SS mice compared to control AA mice. SS-vs-AA_DOWN: genes downregulated in control SS mice compared to control AA mice. SS-vs-AA_Stress_UP: genes enriched in SS mice exposed to RSD compared to AA mice exposed to RSD. SS-vs-AA_Stress_DOWN: genes downregulated in SS mice exposed to stress compared to AA mice exposed to stress. SS_Drug_UP: genes enriched in SS mice treated with minocycline compared to control SS mice. SS_Drug_DOWN: genes downregulated in SS mice treated with minocycline compared to control SS mice. SS_Stress_UP: genes enriched in SS mice exposed to RSD compared to control SS mice. SS_Stress_DOWN: genes downregulated in SS mice exposed to RSD compared to control SS mice. SS_Stress_Drug_UP: genes enriched in SS mice exposed to stress and treated with minocycline compared to SS mice exposed to stress only. SS_Stress_Drug_DOWN: genes downregulated in SS mice exposed to stress and treated with minocycline compared to SS mice exposed to stress only. AA_Stress_UP: genes enriched in AA mice exposed to RSD compared to control AA mice. AA_Stress_DOWN: genes downregulated in AA mice exposed to RSD compared to control AA mice. AA_Stress_Drug_UP: genes enriched in AA mice exposed to stress and treated with minocycline compared to AA mice exposed to stress only. AA_Stress_Drug_DOWN: genes downregulated in AA mice exposed to stress and treated with minocycline compared to AA mice exposed to stress only.

We then performed bulk RNA sequencing and gene set enrichment analysis (GSEA) to identify the pathways underlying RSD-linked cognitive deficit and neuroinflammation in SCD (Figure 4). Most of the differentially expressed gene sets in the cortex were involved in cognitive function, synaptic structures, neuronal signaling, and inflammation (Figure 4A). Differences between SS and AA healthy control mice were evident both at baseline and after RSD exposure. We observed that genes connected with blood-brain barrier dysfunction, depressive disorders, and inflammation (*CCR7*, *FOXF1*) were enriched in SS relative to AA mice. SS + RSD mice show enrichment for genes related to neurodegenerative disease processes (*CTNNB1,* [33] *CSF1R*, *VCP* [34–36]), while sirtuins, which prevent aging and neurocognitive diseases [37, 38], were less enriched. In contrast, no significant changes in gene expression were observed in the AA + RSD group. Additionally, *LDLR* expression (associated with long-term memory) was downregulated in the SS + RSD group compared to the AA + RSD group. Together, these findings support our hypothesis that SS mice might have greater susceptibility to the effects of social stressors such as RSD. Furthermore, SS + RSD + minocycline mice showed enrichment for genes related to synaptic structure and plasticity processes (*BDNF*, *ENTPD1*), while genes associated with cell signaling, immune infiltration, and lipid membrane trafficking (*Adora1, ABDH6*, and *Akt1/2*) were downregulated. Notably, excitatory signaling through serotonin receptors (5-HTR 4, 6, and 7), glutamatergic, and dopaminergic synapses was decreased, suggesting minocycline’s role in preventing stress-linked excitotoxicity and neurodegeneration [39, 40]. Pathways linked to inflammation, gliogenesis, and neuronal death were less enriched in SS + RSD + minocycline animals, while sirtuin-related pathways and processes were enriched. In the hippocampus, similar trends were observed (Figure 4B), with genes related to abnormal cerebral vasculature, blood-brain barrier dysfunction, and inflammatory processes being more enriched in SS compared to AA mice. In SS + RSD mice, genes related to brain development (*MAOB*) and neurodegeneration were significantly enriched compared to AA + RSD mice. Furthermore, SS + RSD mice showed significant enrichment of genes negatively associated with forebrain morphogenesis and neurogenesis and positively associated with inflammation. Taken together, our results suggest that minocycline may help prevent imbalances in synaptic activity and functional decline in SS mice exposed to stress.

**FIGURE 5:**
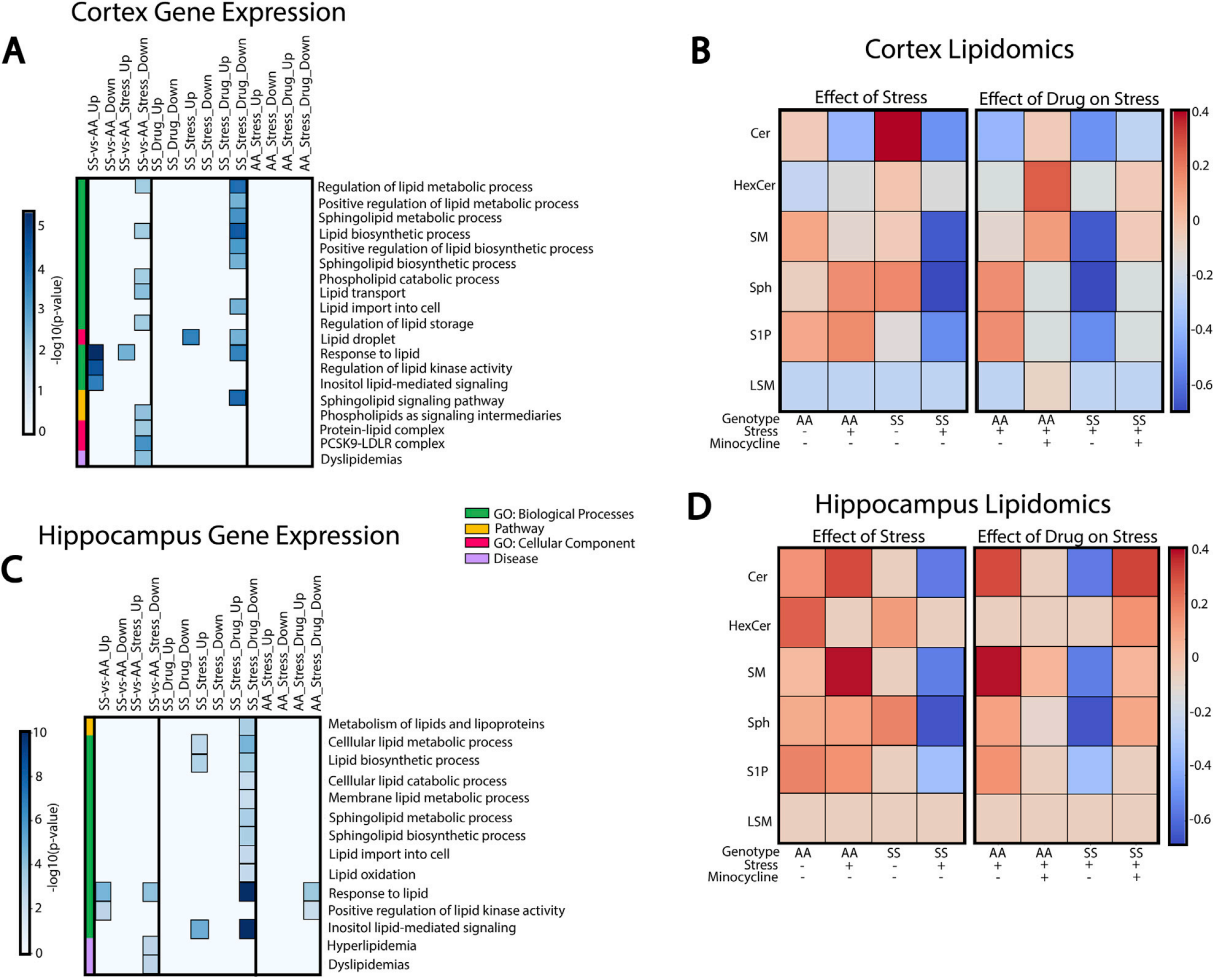
Sphingolipids and lipid metabolism in RSD-mediated inflammation and cognitive impairment. **(A)** Cortex GSEA results of RSD exposure and minocycline-treated mice. **(B)** Mass spectrometry characterization of sphingolipid species found in the cortex. **(C)** Hippocampus GSEA results of RSD exposure and minocycline-treated mice. **(D)** Mass spectrometry characterization of sphingolipid species in the hippocampus.

Because sphingolipids play important roles in neurological function and immune signaling, we investigated their potential connection to neuroinflammation and cognitive deficits induced by social stress in SCD. GSEA analysis was performed to evaluate enrichment of sphingolipid-related pathways in the cortex (Figure 5A) and hippocampus (Figure 5C), while liquid chromatography-mass spectrometry (LC-MS) was used to quantify the concentrations of sphingolipids found in these two brain regions (Figures 5B, D).

In the cortex (Figure 5A), AA and SS mice have significantly different gene expression profiles with and without stress exposure. SS mice exhibit enriched processes related to lipid stimulus responses and lipid kinase activity regulation compared to AA mice. Conversely, processes related to lipid synthesis, metabolism, transport, and storage were downregulated in SS mice. In SS + RSD + minocycline mice, we observed substantial changes in sphingolipid-related pathways and processes. Processes governing lipid metabolism, transport, and storage, including sphingolipids, are downregulated in SS + RSD + minocycline mice compared to SS + RSD mice. Overall, genes associated with sphingolipid signaling and metabolism pathways were also downregulated. In particular, SS + RSD + minocycline mice showed lower expression of genes associated with ceramide (Cer) metabolism, including *de novo* cer synthesis (*Elovl4*, *Slc1a4*) [41], degradation of sphingomyelin (SM) into cer via the salvage pathway (*SMPD1*), and synthesis of ceramide-derived sphingolipids like sphingosine-1-photphate (S1P) (via *Sphk1*) and complex gangliosides (via *ST3GAL2*) [42].

We analyzed LC-MS results to identify a link between pathway enrichment/gene expression and lipidomics profile in the cerebral cortex or hippocampus. In Figure 5B, we found that AA mice had higher levels of Cer and SM and lower levels of hexosylceramide (HexCer), sphingosine (Sph), and S1P compared to AA + RSD mice. Interestingly, SS mice, with or without exposure to RSD, had the highest level of Cer of the two genotypes compared to their AA counterparts. Also, SS mice had higher levels of HexCer, SM, Sph, and S1P compared to SS + RSD mice. This later point may indicate that exposure to RSD/social stress alters the sphingolipid profile by potentially decreasing enzymatic activity in the sphingolipid and thus sphingomyelin biosynthetic and degradation pathways. Minocycline treatment had a significant impact on the sphingolipid profile. Minocycline treatment significantly impacted the sphingolipid profile, with AA + RSD + minocycline mice showing increased Cer, HexCer, SM, and LSM levels and decreased Sph and S1P levels compared to AA + RSD mice. Similarly, SS + RSD + minocycline mice had higher SM, Sph, and S1P levels and lower LSM levels compared to SS + RSD. Overall, these findings suggest that minocycline may restore sphingolipid enzymatic activity perturbed by social stress, which could be one mechanism of its benefit.

Likewise, GSEA and LC-MS analyses of hippocampal tissue showed differential expression of genes involved in critical biological processes between AA and SS mice, as well as between SS + RSD and SS mice (Figure 5C). Specifically, SS + RSD + minocycline mice exhibited downregulation of processes related to lipid metabolism, synthesis, and transport of lipid species (including sphingolipids) and responses to lipid stimuli compared to SS + RSD mice. Notably, several genes responsible for inhibiting (1) *de novo* ceramide synthesis (*ORMDL2*/*ORMDL3*) [43], (2) breaking down lysosomal sphingomyelin to ceramide (*SMPD1*), (3) synthesizing gangliosides from ceramide (*ST3GAL2*/*ST3GAL3*) [42], and (4) converting S1P to sphingosine (*PLPP3*) were significantly less enriched compared to SS + RSD [44]. We noted that some genes involved in the response to lipid stimuli process (*CD38, CX3CR1*, and *TLR2*) and downregulated in SS + RSD + minocycline mice encode surface receptors found on microglia and lymphoid cells [45–48], potentially explaining the reduced neuroinflammation, neurodegeneration, and improved cognitive function observed in the treated mice in our study [48, 49].

As before, we examined the link between gene-set enrichment and concentrations of sphingolipid, this time in the hippocampus. Results from the LC-MS analyses of hippocampal tissue showed contrasting levels compared to the cortex, especially in AA mice, with higher sphingolipid levels (Figure 5D). AA + RSD mice had elevated Cer and SM levels but lower HexCer, Sph, and S1P levels. Also, SS mice had lower levels of Cer, HexCer, SM, and S1P, except Sph, compared to AA mice. SS + RSD mice had reduced levels of all sphingolipids, particularly Sph. AA + RSD + minocycline mice showed decreased levels of Cer, SM, Sph, and S1P. SS + RSD + minocycline mice exhibited higher levels of all sphingolipids compared to SS + RSD mice, except for LSM, which remained consistent across all groups.

## Discussion

In this study, we sought to understand how RSD affects cognitive function (with or without treatment) in male humanized Townes sickle mice compared to control (treated and untreated) mice. Our findings presented above and the online Supplementary Material support our stated hypothesis and show that social stressors (RSD) impair cognitive functions in sickle mice, similar to what was described among children with SCD by King et al. [14, 15] It also provides some mechanistic insight in showing that neuroinflammation and possibly depression of neurogenesis (Figure 3), with a shift towards astrogliogenesis (Supplementary Figure S2), may be among the underlying mechanisms. In our prior work, we showed that 13-month-old Townes sickle mice had more severe cognitive and neurobehavioral deficits and abnormal neuroplasticity [29]. The findings from that study motivated this work in understanding why children with SCD living in a socially stressful environment have more severe manifestations of cognitive deficit. Thus, we additionally showed that minocycline treatment alleviates neuroinflammation, improved neurogenesis and thus, leads to better cognitive and neurobehavioral functions as well as improvement in relevant molecular and cellular phenotypes.

It is known that individuals with SCD experience cognitive and neurobehavioral (anxiety and depression) deficits observed in early childhood, adolescents, and adults [50, 51]. We saw sickle mice exhibit cognitive and neurobehavioral deficits after being exposed to social stress, recapitulating what was described in children with SCD. In these children, it was shown that the presence of cognitive deficit was associated with “biological factors” such as severity of anemia and presence of silent cerebral infarct (SCI) or stroke [52–54]. However, King et al [14, 15] demonstrated more severe evidence of cognitive deficit in children without SCI but who were exposed to social stress in the form of low parental socioeconomic status. This and the report by Andreotti et al. [55], were essentially recapitulated in our study, which showed one or more mechanisms that may underlie the development of cognitive deficit in children with SCD.

In our study, evaluating the hippocampus and dentate gyrus revealed the presence of evidence of neuroinflammation in sickle mice at baseline, i.e., without exposure to RSD. We noted that sickle cell mice exposed to stress had higher densities of “activated microglia” and CD45^+^ “bone-marrow-derived” microglia compared to control mice or sickle cell mice exposed to stress and treated with minocycline. These findings are of particular interest as increased microglia activation or overactive microglia undergoes phenotypical and functional changes, often resulting in increased pro-inflammatory cytokine secretion and increased phagocytosis. These activities have been shown to be involved in the mechanism of cognitive impairment and cause neurobehavioral changes (anxiety and anhedonia) [56–58]. Additionally, studies have shown that peripheral mononuclear cells aka “bone-marrow-derived” microglia, infiltrate the brain parenchyma after psychological stress and further lead to neuroinflammation, anxiety, and memory deficit [59]. Taken together, this suggests that social stress promotes peripheral immune cell infiltration regardless of genotype, but more so in sickle cell, which is already in a pro-inflammatory state. In our study, we did not see increased lymphocyte densities in sickle mice exposed to stress; however, we did note that sickle mice overall, without exposure to RSD, had higher T and B cell densities as well as a higher density of “bone-marrow-derived” microglia compared to AA control mice. These observations support our assertion of a background neuroinflammation in SCD, which was accentuated by exposure to RSD, leading to cognitive deficit. Additionally, recent studies have reported that B cells contribute to neuroinflammation via peripheral immune mechanisms through the production of pro-inflammatory cytokines and antibodies, while effector T cells interaction with microglia can further promote inflammation [60, 61]. This may explain why we observed a higher density of T cells with RSD exposure but did not observe a higher density of B cells. Furthermore, the presence of a higher density of peripheral immune cells in the brain in sickle cell mice indicates their possible role in SCD-related neuroinflammation even in the absence of social stress. We did not adequately examine the presence of T or B cells, for instance, in our prior study; however, it is conceivable to assume they were involved in our observation [29]. Overall, these findings illustrate the potential cellular mechanisms that contribute to cognitive deficits in sickle cell mice exposed to stress and could underlie the observation among SCD patients exposed to social stress, such as lower individual or parental socioeconomic status.

Chronic social stress modulates neurogenesis by decreasing neuron proliferation, resulting in modifications to hippocampal synaptic signaling and plasticity [62]. However, the effect of stress on neurogenesis in SCD is still unknown. In our study, we noted that neural progenitor cells (NPCs) in the dentate gyrus of sickle mice exposed to RSD shifted more (in their differentiation) towards astrogliogenesis as opposed to mature neurons. It has been documented that minocycline improves neurogenesis and mitigates the gliogenic effect of inflammatory cytokines on NPCs [63–66]. This was also observed in our study, where we noted that SS + RSD + minocycline mice had significantly higher densities of adult-born neurons, lower densities of new astrocytes, and lower densities of proinflammatory cells in the hippocampus/DG compared to SS + RSD mice. Intriguingly, minocycline treatment of AA mice led to increased NPC density as well, but not on density of adult-born neurons. The analysis of bulk RNA sequencing and lipidomics supported our other findings that exposure to RSD/social stressors negatively affects the brain, leading to structural remodeling, particularly in SS mice. Treatment with minocycline led to a unique enrichment signature in the cortex of SS mice exposed to stress, where genes associated with cerebral structure remodeling, blood-brain barrier integrity, brain development, neurogenesis, and inflammation were down-regulated in SS + RSD + minocycline mice.

Together, these results support minocycline’s function in preventing neuroinflammation, evidence of neurodegeneration, and cognitive deficit in SS mice exposed to stress and suggest that these might underly the mechanism of social stress-related cognitive deficit in SCD.

## Conclusions and limitations

We have attempted to show some of the underlying mechanisms of how RSD affects cognitive deficits in SCD mice exposed to social stress. We showed that the development of cognitive deficit is in part driven by “activation” of resident immune cells and/or infiltration of peripheral immune cells, astrogliogenesis, changes to lipid metabolism, and the transcriptome. Finally, we demonstrated that treatment with minocycline (which is anti-neuroinflammatory and a sphingomyelinase inhibitor) mitigated the presence of cognitive deficit, possibly by blocking neuroinflammation and shifting NPCs towards neurogenesis. It also supports a favorable lipidomics and transcriptomic profile that promotes neurogenesis and synaptogenesis as well as synaptic plasticity but is anti-excitotoxic and anti-neuroinflammatory.

One limitation of our study is that we used the RSD paradigm, which is likely not representative of the way individuals with sickle cell disease are exposed to social stress in everyday life. Related to this is the fact that this form of stress is more easily carried out in male mice, limiting the conclusions that could be drawn from our study. To the later point, we are now working on a chronic stress model using the social disruption paradigm, which allows us to use both male and female mice. We hope to share the result of this new approach in future publications. Another limitation is the imbalance in the number of mice. There was sample attrition due to mortality; however, this did not confound the direction of the observation.

## Data Availability

The datasets presented in this study can be found in online repositories. The names of the repository/repositories and accession number(s) can be found in the article/Supplementary Material

## References

[1] Steiner CA Miller JL. Statistical brief #21: sickle cell disease patients in U.S. Hospitals. In: Healthcare cost and utilization project. Rockville (MD): Agency for healthcare research and quality. (2004). 21938835

[2] Kato GJ Piel FB Reid CD Gaston MH Ohene-Frempong K Krishnamurti L Sickle cell disease. Nat Rev Dis Primers (2018) 4:18010–22. 10.1038/nrdp.2018.10 29542687

[3] Nader E Romana M Connes P. The red blood cell—inflammation vicious circle in sickle cell disease. Front Immunol (2020) 11:454. 10.3389/fimmu.2020.00454 32231672 PMC7082402

[4] Lettre G. Blocking HbS polymerization in SCD. Cell (2020) 180:819. 10.1016/j.cell.2020.01.019 32142671

[5] DeBaun M Jordan L King A Schatz J Vichinsky E Fox C American Society of Hematology 2020 guidelines for sickle cell disease: prevention, diagnosis, and treatment of cerebrovascular disease in children and adults. Blood Adv (2020) 4:1554–88. 10.1182/bloodadvances.2019001142 32298430 PMC7189278

[6] Heitzer AM Cohen DL Okhomina VI Trpchevska A Potter B Longoria J Neurocognitive functioning in preschool children with sickle cell disease. Pediatr Blood and Cancer (2022) 69:e29531. 10.1002/pbc.29531 PMC9207743 34971013

[7] Heitzer AM Okhomina VI Trpchevska A MacArthur E Longoria J Potter B Social determinants of neurocognitive and academic performance in sickle cell disease. Pediatr Blood and Cancer (2023) 70:e30259. 10.1002/pbc.30259 PMC10339212 36815529

[8] Heitzer AM Hamilton L Stafford C Gossett J Ouellette L Trpchevska A Academic performance of children with sickle cell disease in the United States: a meta-analysis. Front Neurol (2021) 12:786065. 10.3389/fneur.2021.786065 34966350 PMC8711768

[9] Portela GT Butters MA Brooks MM Candra L Rosano C Novelli EM. Comprehensive assessment of cognitive function in adults with moderate and severe sickle cell disease. Am J Hematol (2022) 97:E344–e346. 10.1002/ajh.26643 35749262 PMC9378513

[10] Oluwole O Fertrin KY Kruse-Jarres R. Neurocognitive assessment of adults with sickle cell disease: a descriptive study. Blood (2021) 138:4172. 10.1182/blood-2021-145808

[11] Asnani MR Knight Madden J Reid M Greene L-G Lyew-Ayee P. Socio-environmental exposures and health outcomes among persons with sickle cell disease. PLoS One (2017) 12:e0175260. 10.1371/journal.pone.0175260 28384224 PMC5383275

[12] Khan SA AlSiny F Makki A Ali A AlAnsari I Khan S. Socioeconomic status dependent medical complexities in children with sickle cell disease in Saudi Arabia. Saudi J Biol Sci (2020) 27:1781–7. 10.1016/j.sjbs.2020.03.008 32565696 PMC7296505

[13] Jesus ACS Konstantyner T Lôbo IKV Braga JAP. Características socioeconômicas e nutricionais de crianças e adolescentes com anemia falciforme: uma revisão sistemática. Revista Paulista de Pediatria (2018) 36:491–9. 10.1590/1984-0462/;2018;36;4;00010 30540112 PMC6322809

[14] King AA Rodeghier MJ Panepinto JA Strouse JJ Casella JF Quinn CT Silent cerebral infarction, income, and grade retention among students with sickle cell anemia. Am J Hematol (2014) 89:E188–192. 10.1002/ajh.23805 25042018 PMC4261188

[15] King AA Strouse JJ Rodeghier MJ Compas BE Casella JF McKinstry RC Parent education and biologic factors influence on cognition in sickle cell anemia. Am J Hematol (2014) 89:162–7. 10.1002/ajh.23604 24123128 PMC4310566

[16] Finnell JE Wood SK. Putative inflammatory sensitive mechanisms underlying risk or resilience to social stress. Front Behav Neurosci (2018) 12:240. 10.3389/fnbeh.2018.00240 30416436 PMC6212591

[17] Wohleb ES Powell ND Godbout JP Sheridan JF. Stress-induced recruitment of bone marrow-derived monocytes to the brain promotes anxiety-like behavior. The J Neurosci (2013) 33:13820–33. 10.1523/jneurosci.1671-13.2013 23966702 PMC3755721

[18] McKim DB Niraula A Tarr AJ Wohleb ES Sheridan JF Godbout JP. Neuroinflammatory dynamics underlie memory impairments after repeated social defeat. J Neurosci (2016) 36:2590–604. 10.1523/JNEUROSCI.2394-15.2016 26937001 PMC4879207

[19] Wohleb ES Patterson JM Sharma V Quan N Godbout JP Sheridan JF. Knockdown of interleukin-1 receptor type-1 on endothelial cells attenuated stress-induced neuroinflammation and prevented anxiety-like behavior. J Neurosci (2014) 34:2583–91. 10.1523/JNEUROSCI.3723-13.2014 24523548 PMC3921428

[20] Lee JY Jin HK Bae J-s. Sphingolipids in neuroinflammation: a potential target for diagnosis and therapy. BMB Rep (2020) 53:28–34. 10.5483/bmbrep.2020.53.1.278 31818364 PMC6999823

[21] Asle-Rousta M Oryan S Ahmadiani A Rahnema M. Activation of sphingosine 1-phosphate receptor-1 by SEW2871 improves cognitive function in Alzheimer's disease model rats. EXCLI J (2013) 12:449–61. 26417237 PMC4566907

[22] Brodowicz J Przegalinski E Muller CP Filip M. Ceramide and its related neurochemical networks as targets for some brain disorder therapies. Neurotoxicity Res (2018) 33:474–84. 10.1007/s12640-017-9798-6 PMC5766709 28842833

[23] Efstathopoulos P Kourgiantaki A Karali K Sidiropoulou K Margioris AN Gravanis A Fingolimod induces neurogenesis in adult mouse hippocampus and improves contextual fear memory. Translational psychiatry (2015) 5:e685. 10.1038/tp.2015.179 26795749 PMC5545691

[24] Kanno T Nishizaki T Proia RL Kajimoto T Jahangeer S Okada T Regulation of synaptic strength by sphingosine 1-phosphate in the hippocampus. Neuroscience (2010) 171:973–80. 10.1016/j.neuroscience.2010.10.021 20950672

[25] Saleem M Ratnam Bandaru VV Herrmann N Swardfager W Mielke MM Oh PI Ceramides predict verbal memory performance in coronary artery disease patients undertaking exercise: a prospective cohort pilot study. BMC Geriatr (2013) 13:135. 10.1186/1471-2318-13-135 24330446 PMC3924163

[26] Saleem M Herrmann N Dinoff A Mielke MM Oh PI Shammi P A lipidomics approach to assess the association between plasma sphingolipids and verbal memory performance in coronary artery disease patients undertaking cardiac rehabilitation: a C18:0 signature for cognitive response to exercise. J Alzheimer's Dis (2017) 60:829–41. 10.3233/JAD-161292 28598843 PMC5753402

[27] Ayub M Jin H-K Bae J-s. Novelty of sphingolipids in the central nervous system physiology and disease: focusing on the sphingolipid hypothesis of neuroinflammation and neurodegeneration. Int J Mol Sci (2021) 22:7353. 10.3390/ijms22147353 34298977 PMC8303517

[28] Henriques A Croixmarie V Bouscary A Mosbach A Keime C Boursier-Neyret C Sphingolipid metabolism is dysregulated at transcriptomic and metabolic levels in the spinal cord of an animal model of amyotrophic lateral sclerosis. Front Mol Neurosci (2017) 10:433. 10.3389/fnmol.2017.00433 29354030 PMC5758557

[29] Hardy RA Rached NA Jones JA Archer DR Hyacinth HI. Role of age and neuroinflammation in the mechanism of cognitive deficits in sickle cell disease. Exp Biol Med (2021) 246:106–20. 10.1177/1535370220958011 PMC7797997 32962408

[30] Ryan TM Ciavatta DJ Townes TM. Knockout-transgenic mouse model of sickle cell disease. Science (1997) 278:873–6. 10.1126/science.278.5339.873 9346487

[31] Jonas P Lisman J. Structure, function, and plasticity of hippocampal dentate gyrus microcircuits. Front Neural Circuits (2014) 8:107. 10.3389/fncir.2014.00107 25309334 PMC4159971

[32] Ben Menachem-Zidon O Goshen I Kreisel T Ben Menahem Y Reinhartz E Ben Hur T Intrahippocampal transplantation of transgenic neural precursor cells overexpressing interleukin-1 receptor antagonist blocks chronic isolation-induced impairment in memory and neurogenesis. Neuropsychopharmacology (2008) 33:2251–62. 10.1038/sj.npp.1301606 17987063

[33] Cheng M Luk H-m Lo IF Chung BH. CTNNB1 neurodevelopmental disorder (1993). 35593792

[34] Hu B Duan S Wang Z Li X Zhou Y Zhang X Insights into the role of CSF1R in the central nervous system and neurological disorders. Front Aging Neurosci (2021) 13:789834. 10.3389/fnagi.2021.789834 34867307 PMC8634759

[35] Wong TH Pottier C Hondius DC Meeter LH Van Rooij JG Melhem S Three VCP mutations in patients with frontotemporal dementia. J Alzheimer's Dis (2018) 65:1139–46. 10.3233/jad-180301 30103325

[36] Smith C Badadani M Nalbandian A Dec E Vesa J Donkervoort S Valosin-containing protein (VCP) disease and familial Alzheimer’s disease: contrasts and overlaps. In: The clinical spectrum of alzheimer's disease-the charge toward comprehensive diagnostic and therapeutic strategies. IntechOpen (2011). 10.5772/18811

[37] Bonda DJ Lee H-g Camins A Pallàs M Casadesus G Smith MA The sirtuin pathway in ageing and Alzheimer disease: mechanistic and therapeutic considerations. The Lancet Neurol (2011) 10:275–9. 10.1016/s1474-4422(11)70013-8 21349442 PMC3163839

[38] Madeo F Carmona-Gutierrez D Hofer SJ Kroemer G. Caloric restriction mimetics against age-associated disease: targets, mechanisms, and therapeutic potential. Cell Metab (2019) 29:592–610. 10.1016/j.cmet.2019.01.018 30840912

[39] Andrews M Tousi B Sabbagh MN. 5HT6 antagonists in the treatment of Alzheimer’s dementia: current progress. Neurol Ther (2018) 7:51–8. 10.1007/s40120-018-0095-y 29728891 PMC5990506

[40] Ciranna L. Serotonin as a modulator of glutamate-and GABA-mediated neurotransmission: implications in physiological functions and in pathology. Curr neuropharmacology (2006) 4:101–14. 10.2174/157015906776359540 PMC2430669 18615128

[41] Esch BM Limar S Bogdanowski A Gournas C More T Sundag C Uptake of exogenous serine is important to maintain sphingolipid homeostasis in *Saccharomyces cerevisiae* . PLoS Genet (2020) 16:e1008745. 10.1371/journal.pgen.1008745 32845888 PMC7478846

[42] Olsen AS Færgeman NJ. Sphingolipids: membrane microdomains in brain development, function and neurological diseases. Open Biol (2017) 7:170069. 10.1098/rsob.170069 28566300 PMC5451547

[43] Bugajev V Halova I Demkova L Cernohouzova S Vavrova P Mrkacek M ORMDL2 deficiency potentiates the ORMDL3-dependent changes in mast cell signaling. Front Immunol (2020) 11:591975. 10.3389/fimmu.2020.591975 33643282 PMC7905224

[44] Kono M Hoachlander-Hobby LE Majumder S Schwartz R Byrnes C Zhu H Identification of two lipid phosphatases that regulate sphingosine-1-phosphate cellular uptake and recycling. J Lipid Res (2022) 63:100225. 10.1016/j.jlr.2022.100225 35568252 PMC9213771

[45] Levy A Bercovich-Kinori A Alexandrovich AG Tsenter J Trembovler V Lund FE CD38 facilitates recovery from traumatic brain injury. J neurotrauma (2009) 26:1521–33. 10.1089/neu.2008.0746 19257806 PMC2864472

[46] Morandi F Horenstein AL Costa F Giuliani N Pistoia V Malavasi F. CD38: a target for immunotherapeutic approaches in multiple myeloma. Front Immunol (2018) 9:2722. 10.3389/fimmu.2018.02722 30546360 PMC6279879

[47] Liu H Wang X Chen L Chen L Tsirka SE Ge S Microglia modulate stable wakefulness via the thalamic reticular nucleus in mice. Nat Commun (2021) 12:4646–16. 10.1038/s41467-021-24915-x 34330901 PMC8324895

[48] Fiebich BL Batista CRA Saliba SW Yousif NM de Oliveira ACP. Role of microglia TLRs in neurodegeneration. Front Cell Neurosci (2018) 12:329. 10.3389/fncel.2018.00329 30333729 PMC6176466

[49] Hickman SE Allison EK Coleman U Kingery-Gallagher ND El Khoury J. Heterozygous CX3CR1 deficiency in microglia restores neuronal β-amyloid clearance pathways and slows progression of alzheimer's like-disease in PS1-APP mice. Front Immunol (2019) 10:2780. 10.3389/fimmu.2019.02780 31849963 PMC6900980

[50] Thompson RJ Jr Gustafson KE Bonner MJ Ware RE. Neurocognitive development of young children with sickle cell disease through three years of age. J Pediatr Psychol (2002) 27:235–44. 10.1093/jpepsy/27.3.235 11909931

[51] Graves JK Hodge C Jacob E. Depression, anxiety, and quality of life in children and adolescents with sickle cell disease. Pediatr Nurs (2016) 42:113–44. 27468512

[52] DeBaun MR Armstrong FD McKinstry RC Ware RE Vichinsky E Kirkham FJ. Silent cerebral infarcts: a review on a prevalent and progressive cause of neurologic injury in sickle cell anemia. Blood (2012) 119:4587–96. 10.1182/blood-2011-02-272682 22354000 PMC3367871

[53] DeBaun MR Gordon M McKinstry RC Noetzel MJ White DA Sarnaik SA Controlled trial of transfusions for silent cerebral infarcts in sickle cell anemia. N Engl J Med (2014) 371:699–710. 10.1056/NEJMoa1401731 25140956 PMC4195437

[54] Schatz J Brown RT Pascual JM Hsu L DeBaun MR. Poor school and cognitive functioning with silent cerebral infarcts and sickle cell disease. Neurology (2001) 56:1109–11. 10.1212/wnl.56.8.1109 11320190

[55] Andreotti C King AA Macy E Compas BE DeBaun MR. The association of cytokine levels with cognitive function in children with sickle cell disease and normal MRI studies of the brain. J child Neurol (2015) 30:1349–53. 10.1177/0883073814563140 25512362 PMC4466214

[56] Zhang D Li S Hou L Jing L Ruan Z Peng B Microglial activation contributes to cognitive impairments in rotenone-induced mouse Parkinson's disease model. J Neuroinflammation (2021) 18:4. 10.1186/s12974-020-02065-z 33402167 PMC7786472

[57] Wang Q Chen G Schindler SE Christensen J McKay NS Liu J Baseline microglial activation correlates with brain amyloidosis and longitudinal cognitive decline in alzheimer disease. Neurol - Neuroimmunology Neuroinflammation (2022) 9:e1152. 10.1212/NXI.0000000000001152 35260470 PMC8906187

[58] Schramm E Waisman A. Microglia as central protagonists in the chronic stress response. Neurol - Neuroimmunology Neuroinflammation (2022) 9:e200023. 10.1212/NXI.0000000000200023 36357946 PMC9453699

[59] Wohleb ES Powell ND Godbout JP Sheridan JF. Stress-induced recruitment of bone marrow-derived monocytes to the brain promotes anxiety-like behavior. J Neurosci (2013) 33:13820–33. 10.1523/JNEUROSCI.1671-13.2013 23966702 PMC3755721

[60] Ahn JJ Abu-Rub M Miller RH. B cells in neuroinflammation: new perspectives and mechanistic insights. Cells (2021) 10:1605. 10.3390/cells10071605 34206848 PMC8305155

[61] Schetters STT Gomez-Nicola D Garcia-Vallejo JJ Van Kooyk Y. Neuroinflammation: microglia and T cells get ready to tango. Front Immunol (2017) 8:1905. 10.3389/fimmu.2017.01905 29422891 PMC5788906

[62] Smith KE Pollak SD. Early life stress and development: potential mechanisms for adverse outcomes. J Neurodevelopmental Disord (2020) 12:34. 10.1186/s11689-020-09337-y PMC7745388 33327939

[63] Giri PK Lu Y Lei S Li W Zheng J Lu H Pretreatment with minocycline improves neurogenesis and behavior performance after midazolam exposure in neonatal rats. Neuroreport (2018) 29:153–9. 10.1097/wnr.0000000000000937 29256977 PMC5802258

[64] Kohman RA Bhattacharya TK Kilby C Bucko P Rhodes JS. Effects of minocycline on spatial learning, hippocampal neurogenesis and microglia in aged and adult mice. Behav Brain Res (2013) 242:17–24. 10.1016/j.bbr.2012.12.032 23274840 PMC3725815

[65] Mattei D Djodari-Irani A Hadar R Pelz A de Cossio LF Goetz T Minocycline rescues decrease in neurogenesis, increase in microglia cytokines and deficits in sensorimotor gating in an animal model of schizophrenia. Brain Behav Immun (2014) 38:175–84. 10.1016/j.bbi.2014.01.019 24509090

[66] Vay SU Blaschke S Klein R Fink GR Schroeter M Rueger MA. Minocycline mitigates the gliogenic effects of proinflammatory cytokines on neural stem cells. J Neurosci Res (2016) 94:149–60. 10.1002/jnr.23686 26525774

