# Supplementary material for: Neuroinflammation underlies the development of social stress induced cognitive deficit in male sickle cell mice
Source: Exp Biol Med (Maywood). 2024 Nov 19;249:10361. doi: 10.3389/ebm.2024.10361 (PMC11612828; doi:10.3389/ebm.2024.10361)
Supplement: Supplementary file 1 [file DataSheet1.docx]

**Online Supplemental**

**S’Dravious A. DeVeaux, MS^1,2,^**^†^**, Sofiya Vyshnya, BS^1,2,^**^†^**, Katherine Propsom, BS^3^, Oluwabukola T. Gbotosho, PhD^3^, Asem S. Singh, PhD^3^, Robert Z. Horning, MSc^3^, Mihika Sharma, MS^4^, Anil G. Jegga, DVM, MRes^4^, Edward A. Botchwey, PhD^1,2^, Hyacinth I. Hyacinth, MD, PhD, MPH^3^**

**Methods**

*Repeated Social Defeat (RSD):* RSD was performed as previously described in the literature ^1^ to model sociological stress. In brief, an aggressive male intruder mouse was introduced into cages of established male cohorts for two hours, between 5:00 P.M. and 7:00 P.M., for six consecutive nights. Each cohort contained three HbSS or HbAA mice. If the intruder did not initiate an attack or was attacked by a resident mouse within 5–10 minutes, a new intruder would be introduced. During each 2-hour cycle, we observed submissive behavior, including upright posture, fleeing, and crouching, to ensure defeat of the resident mice. At the end of the 2-hour period, the intruder mouse was removed from the cage, and the residents were undisturbed until the beginning of the next 2-hour cycle on the following day. No intruder mice were introduced to control mice cages.

*Minocycline treatment*: Minocycline (Sigma-Aldrich) was administered orally in drinking water, and its effects on neuroinflammation, neuronal morphology and plasticity, and brain lipidomic on genotype were examined. Minocycline solutions were prepared fresh daily and administered in opaque sipper bottles because minocycline is photosensitive. To adjust administration dosages to 90 mg/kg based on drinking pattern, mice and water bottles were weighed daily. Oral minocycline treatment began 1 day before the beginning of RSD and was terminated on the last day of RSD, which is approximately 24 hours before cognitive and neurobehavioral evaluation.

*Open field test and novel object recognition:* The open field test and novel object recognition (NOR) test were conducted as part of the same experiment. The open field test measures anxiety-like and depressive behaviors in mice,^2^ while the NOR test is used to evaluate hippocampus-based episodic memory and learning.^3-5^ Both tests were conducted in an open field arena made of plexiglass measuring 12” x 12” x 15” (L x W x H). The details of how these behavioral tests are conducted have been published by our lab here^6^ as well as by others.^3,5,7-9^ Briefly, mice were allowed 2-3 days of habituation, where they were allowed to explore the arena for 5 minutes every day. On the 3rd or 4th day, the mice were trained by placing two identical test objects in the enclosure and allowing the mice to explore them for 5 minutes. Following training, the mice were returned to their home cages for about 30 minutes. After this delay, the learning and memory of the mice were tested by replacing one of the test objects used during training with a novel object in the open field.

*Fear conditioning tests:* The fear conditioning tests are used to evaluate associative memory in mice by measuring their ability to form and retain associations between aversive experiences and environmental cues. The tests were performed over a 3-day period, as previously described.^4^ Briefly, on the first day, mice were placed in the fear conditioning chamber and allowed to explore for 3 minutes. Afterwards, mice were presented with 3 pairs of conditioned and unconditioned stimuli. In each pair, the conditioned stimulus (CS) consisted of a 20-second 85 dB tone, and the unconditioned stimulus (US) consisted of a 2-second 0.5 mA electric shock to the footpad. One minute after the last CS-US pair was presented, the mice were returned to their home cages. On the second day, mice underwent a contextual fear conditioning test. The animals were returned to the fear conditioning chamber, but this time no shock was delivered. Freezing behavior was assessed over a 9-minute period. The percent of each minute that mice spent frozen was recorded. On the third day, mice underwent a cued fear conditioning test. The animals were placed in a new environment and allowed to explore for 2 minutes. Afterwards, the CS was presented every minute for 9 minutes, and freezing behavior was recorded every minute. In both tests, freezing indicates a memory for either the context in which the shock was delivered or the association between the tone and the shock. More freezing indicates better associative learning and memory.^4^

*Immunohistochemistry and digital image analysis for IBA-1 and CD45:* After the cognitive and behavioral testing, mice were brought back to their home cages and sacrificed the next day. Brain samples were collected after transcardial perfusion with sterile phosphate buffered saline (PBS), pH 7.4, followed by 4% formaldehyde in PBS. Brains were post-fixed in 4% formaldehyde for 24 h and then transferred to 0.1% sodium azide in PBS if not immediately sectioned and stored at 4°C. Fixed brains were sectioned (50µm) using a vibratome (1200S Leica Microsystems). Brain regions within the hippocampus were identified by reference markers in accordance with the stereotaxic mouse brain atlas.^7^ To label for IBA-1 or CD45, sections that were stored in azide were washed in PBS and incubated overnight at room temperature in a primary antibody cocktail containing rabbit anti-mouse Iba-1 (1:1000; Wako Chemicals), rat anti-mouse CD45 (1:500; Abcam), and guinea pig anti-mouse NeuN, diluted in an antibody diluting solution (containing 0.1% azide, 2% Triton-x-100, and 10% normal goat serum in PBS). Then sections were washed in PBS and incubated with a fluorochrome-conjugated secondary antibody (Alexa Fluor 488, 594, and 750, respectively). Sections were then mounted on glass slides, cover-slipped with Fluoromount G (Beckman Coulter), and stored at 4°C after drying. Images (z-stacks) of the dentate gyrus on either side in all 3 fluorochrome color channels were acquired at 20X magnification using an SP8 confocal microscope (Leica Microsystems), projected using maximum intensity projection, and then analyzed using NIH ImageJ software. CD45 cells with positive labeling were counted in each dentate gyrus section; the dentate gyrus is a component of the hippocampus. IBA-1 labeling was analyzed using an established and published digital image analysis system.^8^ In brief, a threshold for positive labeling was determined for each image that included all cell bodies and processes but excluded background staining. Data were processed by densitometric scanning of the threshold targets using NIH ImageJ software. The proportional area was reported as the average percentage area in the positive threshold for all representative images.^10^

*Statistical analysis*: statistical analysis was carried out using a combination of one-way and two-way (using a mixed effect model) analysis of variance (ANOVA), with the additional use of appropriate post-hoc analysis when the result of the ANOVA shows the presence of a significant difference across the groups. To minimize the likelihood of a type 2 error, we only performed biologically meaningful comparisons, i.e., we compared AA+RSD+minocycline to SS+RSD+minocycline to evaluate genotype effect, but to SS+RSD, where both genotype and treatment are different. Another example is that we also compared SS+RSD+minocycline to SS+RSD to evaluate the impact of minocycline in SS mice. However, we did not compare SS+RSD+minocycline to SS, as there is a difference in exposure and treatment. This approach meant that we limit the number of comparisons as well as the loss of power and thus the risk of committing type 2 error.

*Sphingolipid Extraction and LC-MS/MS Analysis:*

The details of the protocol used for sphingolipid extraction have been described elsewhere.^11^ Briefly, hippocampal and cortical tissues were homogenized in PBS. Each homogenate was divided into two aliquots. The first aliquot underwent sphingolipid extraction, while the second aliquot was used for total protein quantification with the BCA protein assay. The first aliquot was further divided between long-chain bases (LCBs) and complex sphingolipids (CSLs) extractions. Samples designated for LCB extraction were suspended in a 2:1 methanol:methylene chloride solution, while the samples designated for CSL extraction were suspended in a 2:1 methanol:chloroform solution. Internal standard mixture (Avanti Polar Lipids) was then added to each sample, and samples were incubated overnight. Afterwards, the CSL samples underwent base catalysis for 2 hours before being neutralized. The LCB samples were transferred to new glass tubes, leaving behind tissue debris, and 2:1 methanol:methylene chloride extraction solvent was added to the original glass tubes. Then, tubes were centrifuged to collect residual LCBs from the debris, and the extraction solvent (containing lipids) was transferred to the new glass tube. For the CSL samples, the extraction solvent consisted of water added to the 2:1 methanol:chloroform mixture, allowing for aqueous-organic phase separation. The CSL samples were centrifuged, and the bottom organic layer (containing lipids) was transferred to new glass tubes, leaving behind tissue debris. The extraction solvent was again added to the residual tissue debris, and the samples were centrifuged a second time. The bottom organic phase was once again collected. Finally, the organic solvents from both LCB and CSL samples were subsequently removed by vacuum drying overnight in a Savant SpeedVac (Thermo Fisher). The dried lipids were stored in a -20 C freezer until analysis.

*Analysis of RNA Sequencing, Data Analysis and GO Analysis:*

Directional polyA RNA-seq was performed by the Genomics, Epigenomics, and Sequencing Core at the University of Cincinnati using established protocols as previously mentioned.^12,13^ To summarize, the quality of total RNA was QC analyzed by a Bioanalyzer (Agilent, Santa Clara, CA). To isolate polyA RNA for library preparation, the NEBNext Poly(A) mRNA Magnetic Isolation Module (New England BioLabs, Ipswich, MA) was used with 500 ng of good-quality total RNA as input. The polyA RNA was enriched using the SMARTer Apollo automated NGS library prep system (Takara Bio USA, Mountain View, CA). Next, the NEBNext Ultra II Directional RNA Library Prep Kit (New England BioLabs) was used for library preparation under PCR cycle number of 9. After library QC and quantification via real-time qPCR (NEBNext Library Quant Kit, New England BioLabs), individually indexed libraries were proportionally pooled and sequenced using the NextSeq 550 sequencer (Illumina, San Diego, CA) under the sequencing setting of single read 1x85 bp to generate about 25M reads. After sequencing, fastq files for downstream data analysis were automatically generated via Illumina BaseSpace Sequence.

The raw sequencing reads were aligned to GRCm39 (mm39) using STAR.^14^ The number of reads that were aligned to each gene was obtained while mapping using the GENCODE gene annotation. Then we used edgeR^15^ to identify differentially expressed genes. For each genotype (“AA” or “SS”) in each tissue (hippocampus, cortex), we included all samples in a model and compared the two “exposure” groups (RSD vs. no RSD) and then the “treatment” groups (minocycline treatment vs. no minocycline treatment) in a pairwise fashion. We also compared genotypes; however, we ensured that only biologically meaningful comparisons were performed.

*Functional enrichment analysis and visualization*:

We used ToppFun^16^ and ToppCluster^17^ applications to perform functional enrichment analysis of differentially expressed genes (DEGs) in various groups. Genes differentially expressed in different comparisons were input into the ToppFun/ToppCluster for gene ontology (GO) and biological pathway annotation, with Benjamini and Hochberg’s correction for significance testing set at p value <0.05. To visualize the enriched biological processes and pathways, we used Cytoscape.v3.0.2^18^ (for network representation) and Morpheus (https://software.broadinstitute.org/morpheus/; for heatmap representation) applications.

The raw and processed RNA sequencing data along with the metadata have been uploaded to GEO with the accession **GSE252778**.

**Results**

**
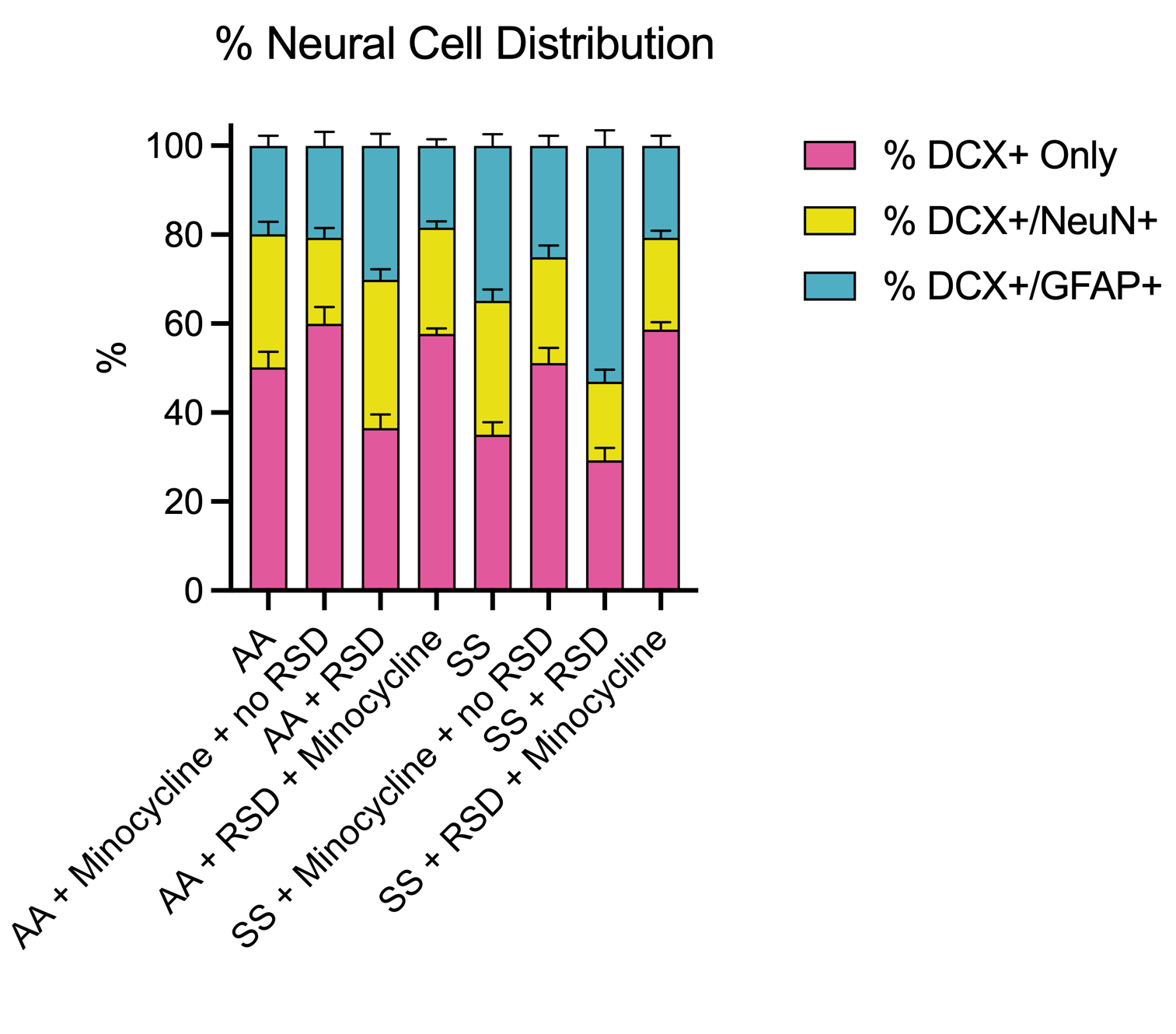
**

**Supplemental Fig.** Hippocampal neural cell distribution in sickle and AA mice.

In **Supplementary Fig. 1**, we assessed the percent distribution of neural cells to evaluate how RSD and minocycline treatment influence neural cell distribution. At baseline, AA mice had 50.1% of DCX+ neural progenitor cells (NPCs), while AA+minocycline mice had approximately 59.9% of NPCs. We observed a lower percent of NPCs in AA+RSD mice (36.5%); however, AA+RSD+minocycline mice had an increase in NPCs (57.7%). SS mice had a lower percentage of NPCs (35.1%) compared to their AA counterparts, and treating SS mice with minocycline increased NPCs percent to 51.2%. SS+RSD mice had the lowest percent among all the groups, with only 29.3% NPCs. Similar to AA mice with the same treatment, SS mice exposed to RSD and treated with minocycline had a 58.7% increase in NPCs. Next, we assessed DCX^+^NeuN^+^ neuron distribution. In AA control mice, we saw a 30.0% neuron distribution; however, treatment with minocycline decreased the neuron percent to 19.4%. AA+RSD mice had 33.4% neurons, while treatment with minocycline decreased this to 23.9%. Comparable to AA control mice, SS mice had 30.0% neurons. This fraction was lower in SS+minocycline mice (23.7%). SS+RSD mice had 17.6% of neurons, and treatment with minocycline increased this to 20.6% of neurons. Lastly, we looked at DCX^+^GFAP^+^ astrocyte distribution. We observed that baseline AA mice had a similar percentage of astrocytes with AA+minocycline mice. AA+RSD mice had 30.1% astrocytes; however, a decrease of astrocytes (18.4%) was noted when AA+RSD mice were treated with minocycline. SS control mice had 34.9% astrocytes, while SS+minocycline mice had 25.1% astrocytes. SS+RSD mice had 53.1% astrocytes, and treatment with minocycline reduced this to 20.7%. This data suggests that RSD decreases NPCs in both AA and SS mice while increasing astrocyte. Additionally, treating mice exposed to stress with minocycline caused a decrease in astrocytes and an increase in NPCs. These results suggest that minocycline treatment may prevent NPC differentiation into astrocytes.

**References**

1. Golden SA, Covington HE, 3rd, Berton O, Russo SJ. A standardized protocol for repeated social defeat stress in mice. *Nat Protoc*. 2011;6:1183-1191. doi: 10.1038/nprot.2011.361

2. Sadegzadeh F, Sakhaie N, Dehghany R, Adak O, Saadati H. Effects of adolescent administration of fluoxetine on novel object recognition memory, anxiety-like behaviors, and hippocampal brain-derived neurotrophic factor level. *Life sciences*. 2020;260:118338.

3. Darcet F, Mendez-David I, Tritschler L, Gardier AM, Guilloux J-P, David DJ. Learning and memory impairments in a neuroendocrine mouse model of anxiety/depression. *Frontiers in behavioral neuroscience*. 2014;8:136.

4. Hardy RA, Rached NA, Jones JA, Archer DR, Hyacinth HI. Role of age and neuroinflammation in the mechanism of cognitive deficits in sickle cell disease. *Experimental Biology and Medicine*. 2021;246:106-120. doi: 10.1177/1535370220958011

5. Moore SJ, Deshpande K, Stinnett GS, Seasholtz AF, Murphy GG. Conversion of short-term to long-term memory in the novel object recognition paradigm. *Neurobiology of learning and memory*. 2013;105:174-185.

6. Hardy RA, Rached NA, Jones JA, Archer DR, Hyacinth HI. Role of age and neuroinflammation in the mechanism of cognitive deficits in sickle cell disease. *Experimental biology and medicine*. 2021;246:106-120. doi: 10.1177/1535370220958011

7. Paxinos G, Franklin KB. *The mouse brain in stereotaxic coordinates*. Gulf Professional Publishing; 2004.

8. Donnelly DJ, Gensel JC, Ankeny DP, van Rooijen N, Popovich PG. An efficient and reproducible method for quantifying macrophages in different experimental models of central nervous system pathology. *J Neurosci Methods*. 2009;181:36-44. doi: 10.1016/j.jneumeth.2009.04.010

9. Ferreira TA, Blackman AV, Oyrer J, Jayabal S, Chung AJ, Watt AJ, Sjöström PJ, Van Meyel DJ. Neuronal morphometry directly from bitmap images. *Nature methods*. 2014;11:982-984.

10. Wohleb ES, Powell ND, Godbout JP, Sheridan JF. Stress-Induced Recruitment of Bone Marrow-Derived Monocytes to the Brain Promotes Anxiety-Like Behavior. *The Journal of Neuroscience*. 2013;33:13820-13833. doi: 10.1523/jneurosci.1671-13.2013

11. DeVeaux SA, Ogle ME, Vyshnya S, Chiappa NF, Leitmann B, Rudy R, Day A, Mortensen LJ, Kurtzberg J, Roy K, et al. Characterizing human mesenchymal stromal cells' immune-modulatory potency using targeted lipidomic profiling of sphingolipids. *Cytotherapy*. 2022;24:608-618. doi: 10.1016/j.jcyt.2021.12.009

12. Rapp SJ, Dershem V, Zhang X, Schutte SC, Chariker ME. Varying Negative Pressure Wound Therapy Acute Effects on Human Split-Thickness Autografts. *J Burn Care Res*. 2020;41:104-112. doi: 10.1093/jbcr/irz122

13. Walsh KB, Zhang X, Zhu X, Wohleb E, Woo D, Lu L, Adeoye O. Intracerebral Hemorrhage Induces Inflammatory Gene Expression in Peripheral Blood: Global Transcriptional Profiling in Intracerebral Hemorrhage Patients. *DNA Cell Biol*. 2019;38:660-669. doi: 10.1089/dna.2018.4550

14. Dobin A, Davis CA, Schlesinger F, Drenkow J, Zaleski C, Jha S, Batut P, Chaisson M, Gingeras TR. STAR: ultrafast universal RNA-seq aligner. *Bioinformatics*. 2013;29:15-21. doi: 10.1093/bioinformatics/bts635

15. Robinson MD, McCarthy DJ, Smyth GK. edgeR: a Bioconductor package for differential expression analysis of digital gene expression data. *Bioinformatics*. 2010;26:139-140. doi: 10.1093/bioinformatics/btp616

16. Chen J, Bardes EE, Aronow BJ, Jegga AG. ToppGene Suite for gene list enrichment analysis and candidate gene prioritization. *Nucleic acids research*. 2009;37:W305-311. doi: 10.1093/nar/gkp427

17. Kaimal V, Bardes EE, Tabar SC, Jegga AG, Aronow BJ. ToppCluster: a multiple gene list feature analyzer for comparative enrichment clustering and network-based dissection of biological systems. *Nucleic acids research*. 2010;38:W96-102. doi: 10.1093/nar/gkq418

18. Shannon P, Markiel A, Ozier O, Baliga NS, Wang JT, Ramage D, Amin N, Schwikowski B, Ideker T. Cytoscape: a software environment for integrated models of biomolecular interaction networks. *Genome research*. 2003;13:2498-2504. doi: 10.1101/gr.1239303
